# Rates of periprosthetic infection and surgical revision in Beijing (China) between 2014 and 2016: a retrospective multicenter cross-sectional study

**DOI:** 10.1186/s13018-019-1520-3

**Published:** 2019-12-26

**Authors:** Hui-ming Peng, Long-chao Wang, Ji-ying Cheng, Yi-xin Zhou, Hua Tian, Jian-hao Lin, Wan-shou Guo, Yuan Lin, Tie-bing Qu, Ai Guo, Yong-ping Cao, Xi-sheng Weng

**Affiliations:** 1 Department of Orthopedics, Peking Union Medical College Hospital, CAMS & PUMC, No.1 Shuaifuyuan, Wangfujing, Dongcheng District, Beijing, 100730 China; 2 Department of Orthopedics, Chinese PLA General Hospital, Beijing, 100853 China; 3 Department of Orthopedics, Beijing Jishuitan Hospital, Beijing, 100035 China; 4 Department of Orthopedics, Peking University Third Hospital, Beijing, 100083 China; 5 Department of Orthopedics, Peking University People’s Hospital, Beijing, 100044 China; 6 Department of Orthopedics, Sino-Japanese Friendship Hospital, Beijing, 100029 China; 7 Department of Orthopedics, Beijing Chaoyang Hospital, Capital Medical University, Beijing, 100020 China; 8 Department of Orthopedics, China Rehabilitation Research Center Beijing Boai Hospital, Beijing, 100068 China; 9 Department of Orthopedics, Beijing Friendship Hospital, Capital Medical University, Beijing, 100050 China; 10 Department of Orthopedics, Peking University First Hospital, Beijing, 100034 China

**Keywords:** Arthroplasty, Hip and knee replacement, Infection, Revision

## Abstract

**Background:**

Periprosthetic joint infection (PJI) is a rare but devastating complication after total joint arthroplasty. There is a paucity of data on the incidence and prevalence of periprosthetic infection in mainland China. This study aimed to analyze the rates of surgical revision after arthroplasty due to PJI and the procedures followed in Beijing, China.

**Methods:**

The study involved a retrospective multicenter cross-sectional survey of patients undergoing revisions for periprosthetic infection after hip/knee arthroplasty at nine hospitals in Beijing from 2014 to 2016. Age, gender, body mass index, primary diagnosis, comorbidity, primary surgery, treatment methods, and post-revision complications were analyzed.

**Results:**

A total of 38,319 hip/knee arthroplasties and 366 (0.96%) revisions for PJI were identified. Of these, 161 (161/14,110; 1.14%) revisions involved hip arthroplasty, whereas 205 (205/24,209; 0.85%) revisions were due to knee arthroplasty. Procedures for revisions of infected hip included 7 (4.3%) cases of open debridement and prosthesis retention, 32 (19.9%) cases of one-stage exchange, 121 (75.2%) cases of two-stage exchange, and 1 (0.007%) case of hip dissection. As for the infected knee, the procedures included 45 (22.0%) cases of open debridement and prosthesis retention, 13 (6.3%) cases of one-stage exchange, 143 (69.8%) cases of two-stage exchange, and 4 (0.02%) cases of knee fusion.

**Conclusions:**

The study found the rates of revision due to PJI to be low. Nonetheless, the incidence of PJI in mainland China could be higher and calls for more elaborate studies in geographically and socioeconomically diverse health institutions.

## Background

Hip or knee arthroplasty is often the last recourse in the treatment of terminal hip and knee diseases. In the UK and the USA, approximately 800,000 joint replacement surgeries are performed annually, and this number is expected to exceed 4 million by the year 2030 [1].

Periprosthetic joint infection (PJI) is a potentially devastating complication after joint replacement. This condition is associated with pain, compromised joint function, prolonged hospitalization, and increased cost of medical care. In some cases, high patient mortality has been reported [2, 3]. Treatment options for periprosthetic infection include debridement, use of antimicrobials, and surgical revision with the aim to restore joint function and relieve pain [4–6]. Surgical revisions and other treatments for PJI are expensive. For example, in the USA alone, the estimated cost of treating periprosthetic infection was 566 million dollars in 2009 and is estimated to reach 1.62 billion dollars by the year 2020 [7].

The reported incidence of periprosthetic infection in the Western populations is 1.2–2.2% [8–10]. On the other hand, there is a paucity of data on the incidence of periprosthetic infection after hip or knee arthroplasty in China. This is likely due to the far more recent development of technical capacity to conduct joint replacement surgery in China. Our study, therefore, aimed to analyze the incidence and management of PJI at 9 main arthroplasty centers in Beijing, China. Data was collected retrospectively for the time period between 2014 and 2016. The nine selected centers are the top referral hospitals in Beijing with high surgical volumes of at least 500 artificial joint replacement surgeries per year.

## Methods

### Patients

Clinical data of patients diagnosed with PJI following hip/knee arthroplasty were collected retrospectively for the time period between 1 January 2014 and 31 December 2016 in the nine health institutions that serve as main arthroplasty centers in Beijing, China. Primary surgeries were hip arthroplasty (including total hip arthroplasty and femoral head replacement) and knee arthroplasty (including total knee arthroplasty and partial knee arthroplasty). The treatment options for periprosthetic infection included open debridement with retention of the prosthesis, one-stage or two-stage revision, and hip disarticulation and hip/knee joint fusion.

### Data collection

Demographic and clinical data included age, gender, body mass index (BMI), primary diagnosis, and preoperative comorbidities. Surgical methods, the occurrence of periprosthetic infection (type of infection, sinus tract involvement, etiological evidence, erythrocyte sedimentation rate, C-reactive protein) after joint replacement, post-infection treatment, and postoperative complications were also recorded. The diagnosis and classification of periprosthetic infection was performed according to the 2012 Musculoskeletal Infectious Society criteria [11].

### Statistical analysis

Statistical analysis was performed using SPSS 20.0. For continuous data, the mean ± standard deviation was computed and data between groups compared using the *t* test. For the classification data, the composition ratio is expressed, and differences between groups established using the chi-square tests. In all cases, a two-sided test was performed; *p* < 0.05 was considered statistically significant.

## Results

### Patient general information

A total of 38,319 cases of hip and/or knee arthroplasty were included in our study. While there was an 8.4% rise in the number of cases from 2014 to 2015, no increase was observed between 2015 and 2016 (Table 1). There were 366 (0.96%) revision cases due to PJI over the duration of the study (Table 1).

**Table 1:** Numbers of hip and knee arthroplasty and periprosthetic infection at the 9 hospitals during 2014–2016

Hospital	2014		2015		2016	
	Hip	Knee	Hip	Knee	Hip	Knee
A	277 (3)	589 (10)	269 (1)	573 (4)	308 (2)	688 (1)
B	1381 (14)	2242 (18)	1508 (10)	2031 (14)	1391 (13)	2178 (14)
C	274 (2)	1115 (8)	306 (2)	1261 (3)	306 (1)	934 (6)
D	500 (9)	1200 (14)	500 (5)	1650 (4)	450 (6)	1700 (8)
E	161 (2)	284 (0)	157 (2)	358 (3)	146 (1)	337 (0)
F	181 (4)	369 (3)	191 (1)	312 (1)	189 (0)	352 (1)
G	58 (1)	65 (3)	119 (1)	136 (1)	126 (0)	141 (2)
H	330 (4)	375 (7)	328 (1)	353 (2)	331 (2)	467 (1)
I	1255 (11)	1457 (19)	1637 (38)	1439 (33)	1431 (25)	1602 (25)
Sum	4417 (50)	7696 (82)	5015 (61)	8114 (75)	4678 (50)	8399 (58)
Total	12,113 (132)		13,128 (136)		13,077 (108)	
	38,319 (366)					

Numbers of periprosthetic infection are indicated in brackets. A, Peking Union Medical College Hospital; B, Chinese PLA General Hospital; C, Beijing Jishuitan Hospital; D, Peking University Third Hospital; E, Peking University People’s Hospital; F, Sino-Japanese Friendship Hospital; G, Beijing Chaoyang Hospital; H, Beijing Friendship Hospital; I, Peking University First Hospital

The mean age of patients who underwent revision surgery due to PJI was 62.3 ± 13.1 years (range 21–86 years) with a slightly higher proportion of females (*n* = 205, 56.0%). On the other hand, the mean BMI of these patients was 25.6 ± 3.8 kg/m^2^ (range 15.6–38.1 kg/m^2^). In these patients, 72 (19.7%) were diagnosed with diabetes prior to the primary surgery. The primary surgery majorly involved unilateral joint replacement (325, 88.8%) with one-stage bilateral joint replacement happening only in 41 patients (11.2%). Of note, the majority of the PJI were due to late infections (318, 86.9%) (Table 2). The overall revision rate after joint replacement was 0.28% (109/38,319) (Table 3).

**Table 2:** Demographic and clinical data of patients with periprosthetic infections

	Hip arthroplasty (*n* = 161)	Knee arthroplasty (*n* = 205)	*p*
Male/female	98/63	63/142	0.000
Age (year)	56.4 ± 15.0	66.7 ± 9.3	0.000
BMI (kg/m^2^)	25.1 ± 4.1	25.9 ± 3.6	0.048
Partial/total arthroplasty	143/17	182/24	0.758
Primary/revision arthroplasty	157/4	201/4	1.000
Diabetes	29/132	43/162	0.479
Primary surgery	47/114	102/103	0.000
Acute/late infection	7/154	41/164	0.000
Infection duration (day)	608.7 ± 849.9	400.0 ± 667.1	0.009
Sinus tract	39/122	52/151	0.644
Positive culture	115/46	141/64	0.583
Single/multiple species	76/39	111/30	0.023
Bacteria resistance	41	28	0.004
ESR (mm/1 h)	41.5 ± 20.6	49.3 ± 23.4	0.001
CRP (mg/L)	24.3 ± 29.8	33.6 ± 47.7	0.031

*BMI* body mass index, *ESR* erythrocyte sedimentation rate, *CRP* C-reactive protein

**Table 3:** Incidence of surgical revision cases in the nine selected hospitals in Beijing, China

Year/hospital	2014			2015			2016			Total		
	Hip	Knee	Total	Hip	Knee	Total	Hip	Knee	Total	Hip	Knee	Total
A	1	6	7	0	2	2	0	1	1	1	9	10
B	4	8	12	0	8	8	2	2	4	6	18	24
C	0	2	2	0	2	2	1	2	3	1	6	7
D	4	5	9	1	5	6	1	2	3	6	12	18
E	0	1	1	0	0	0	0	0	0	0	1	1
F	0	2	2	1	0	1	0	0	0	1	2	3
G	0	1	1	0	1	1	0	0	0	0	2	2
H	0	1	1	0	0	0	0	0	0	0	1	1
I	8	15	23	2	11	13	3	4	7	13	30	43
Total	17	41	58	4	29	33	7	11	18	28	81	109

A, Peking Union Medical College Hospital; B, Chinese PLA General Hospital; C, Beijing Jishuitan Hospital; D, Peking University Third Hospital; E, Peking University People’s Hospital; F, Sino-Japanese Friendship Hospital; G, Beijing Chaoyang Hospital; H, Beijing Friendship Hospital; I, Peking University First Hospital

### Hip replacement

The study recorded 14,110 cases of total hip arthroplasty or femoral head replacement the majority of which were primary hip replacement (13,317, 94.48%) (Table 1). Among the surgical revision cases, 161 cases were associated with infection mostly after the primary replacement. The proportion of infectious hip revision in the hip replacement cases was 1.14% (161/14,110) while the overall revision rate due to PJI after hip replacement in the 9 hospitals was 0.20% (28/14,110) (Table 3).

Primary hip replacement was occasioned by various conditions the most common being femoral neck fracture (*n* = 53, 32.9%) and femoral head necrosis (*n* = 60, 37.3%). Other causes were degenerative osteoarthritis, femoral intertrochanteric fracture, developmental dysplasia of the hip, traumatic arthritis, rheumatoid arthritis, ankylosing spondylitis, tuberculosis, congenital hip dislocation, hip fracture, and septic arthritis. Unknown causes for hip replacement were reported in 11 cases.

Prior to the primary surgery, 29 (18.0%) patients were diagnosed with diabetes and most of the primary surgeries involved unilateral arthroplasty (*n* = 143, 88.8%). Late infections (95.7%) rather than acute infections were the major reasons for surgical revisions. The causes and duration of the infections displayed wide heterogeneity. Over 115 positive cultures were identified from the infected joints with 66% (76/115) arising due to a single species (Table 2). *Staphylococcus* was the leading bacterial species responsible for infection, followed by *Enterococcus*, *Streptococcus*, *Klebsiella*, *Pseudomonas aeruginosa*, *Escherichia coli*, and *Propionibacterium*. Infections with resistant bacteria (*n* = 41) were also documented with some cultures producing negative results (Table 2).

### Knee replacement surgery

Our study identified 24,209 cases of either total or partial knee arthroplasty (Table 1). Most of these cases (*n* = 23,443, 96.8%) were for primary knee replacement. Surgical revision cases due to infections after primary replacement accounted for 201 cases with only 4 cases of infections reported after surgical revision. The overall revision rate due to PJI after knee replacement was 0.33% (81/24,209) (Table 3).

The leading causes of knee replacement were degenerative osteoarthritis (*n* = 163, 79.5%) and femoral neck fracture (*n* = 53, 32.9%); this was followed by traumatic arthritis, rheumatoid arthritis, gouty arthritis, villonodular synovitis, and femoral intertrochanteric fracture. The reasons for knee replacement in 15 cases remained unknown.

Prior to the primary surgery, 43 (21.0%) patients were diagnosed to be diabetic. Similar to hip replacement, partial replacement (*n* = 182, 88.8%) rather than total joint replacement was the dominant surgical procedure. The majority of periprosthetic infections were late infections (80%) with an overall average duration of 400.0 ± 667.1 days (range, 2–4934 days). Positive microbial culture was detected in most of the cases (68.8%), and many of the infections were caused by a single species. *Staphylococcus* was the leading bacterial species followed by *Enterococcus*, *Escherichia*, and *Pseudomonas*.

### Surgical interventions

The surgical interventions for PJI varied and included, for infected hip replacement, 7 (4.3%) cases of open debridement with prosthesis retention, 32 (19.9%) cases of one-stage revisions, 121 (75.2%) cases of two-stage revision, and 1 (0.007%) case of hip *disarticulation*. For infected knee replacement surgery, the procedures included 45 (22.0%) cases of open debridement with prosthesis retention, 13 (6.3%) cases of one-stage revision, 143 (69.8%) cases of two-stage revision, and 4 (0.02%) cases of knee fusion.

## Discussion

This is the first study in China analyzing the incidence of surgical revision due to PJI using a large sample size. Our findings show a decrease in the overall rates of periprosthetic infection and surgical revision after hip and knee replacement at the nine hospitals in Beijing from 2014 to 2016 (0.48 to 0.14%).

Patient-related factors have been implicated to predispose to PJI. Lombardi et al. [12] have proposed that conditions such as obesity and diabetes may increase the risk of periprosthetic infection. On the other hand, Sousa et al. [9] found that the incidence of periprosthetic infection in patients with asymptomatic bacteriuria was significantly higher than that in patients without the condition (4.3% vs. 1.4%). In another case, a prospective study conducted by Peng et al. [13] showed that the application of povidone-iodine for nasal disinfection before orthopedic surgery can effectively reduce the colonization of methicillin-resistant *Staphylococcus aureus*.

The surgical team and surgical procedure have also been identified as risk factors for periprosthetic infection. In their work, Leijtens et al. [14] showed that intraoperative hypothermia is associated with increased risk of infection after total knee arthroplasty. In a different study, Panahi et al. [15] found that the frequency of opening of the operating room door per minute influenced the infection rates. Bacterial contamination of the surgical room was thus linked to an increase in the infection rate. Therefore, the implementation of measures to prevent and control contamination may help to reduce the incidence of periprosthetic infection [16, 17].

There is a consensus that the two-stage revision with antibiotic bone cement spacer is the gold standard for the treatment of chronic periprosthetic infection [18, 19]. This was also the treatment method adopted for most of the patients with periprosthetic infections in our study. However, this surgical procedure has a long course of treatment and is demanding with regard to surgical skills. Furthermore, the procedure is also associated with the risk of serious loss of bone mass during operation and postoperative complications.

The feasibility and efficacy of the one-stage surgical revision for the treatment of periprosthetic infection have been explored [20–23]. It has been shown that the one-stage revision produces comparable treatment outcomes to those of the two-stage revision [24, 25]. In 2015, an international expert consensus recommended the use of the two-stage surgical revision instead of the one-stage revision for patients with severe soft tissue injury, positive culture of resistant bacteria, or sinus tract formation [26].

In the present study, 22.0% of the patients with knee prosthetic infections were treated with open debridement and prosthesis retention. Compared to revision surgery, this procedure has better postoperative knee function and quality of life as more bone mass is retained and there is less risk of postoperative complications [27]. In addition, the procedure is less demanding on the level of surgical skill and takes a shorter operation time. In one study, the reported success rate of knee debridement in treating periprosthetic infection was 44–75% [27, 28]. It has been suggested that sinus tract is a risk factor for the failure of knee debridement and a contraindication for retaining prostheses in managing periprosthetic infection [29]. Marculescu et al. found that patients with sinus tract had a survival rate of only 29% after 2 years of primary surgery, contrasting with the survival rate of 64% in those without sinus tract [30].

In a study by Bradbury et al. and Barberán et al., the success rate of open debridement with prosthesis retention in treating periprosthetic infection patients with a positive culture of resistant bacteria was relatively low (18–28.6%) [29, 31]. Insert exchange in debridement has been shown to be effective in eradicating infection [32–34]. Currently, there is controversy over the appropriate timing of open debridement. The failure rate of open debridement in treating periprosthetic infection significantly increases beyond a certain time after total knee arthroplasty, which varies from 15 days to 1 month in different studies [30, 31, 35].

Although providing useful insights, our study is not devoid of limitations. First, the retrospective nature of the study might introduce bias since our survey was limited to the available documented data. Second, patients who were treated using non-surgical methods for mild infections were not included in our study thereby affecting the composition of the study pool. Third, bacterial culture was only performed for the first and not subsequent episodes of infections. The spectrum of microorganisms responsible for infections may, therefore, not be fully representative of the causative agents of infections. Finally, the sampled patient population was mainly from the northern region of China. It may therefore be impossible to extrapolate these findings to the whole of China.

## Conclusions

The overall rate of periprosthetic infection and surgical revision after hip or knee joint arthroplasty in Beijing, China, was 0.96%. The two-stage revision was the most common surgical method for the treatment of periprosthetic infection, and most infections were caused by *Staphylococcus*. We recommend that surgical methods for the treatment of periprosthetic infection be selected considering a patient’s clinical status and the cost-effectiveness of the procedure.

## Data Availability

All data generated or analyzed during this study are included in this published article.

## Abbreviations

BMI: Body mass index
PJI: Periprosthetic joint infection
